# Arrhythmias and ion channelopathies causing sudden cardiac death in Hispanic/Latino and Indigenous populations

**DOI:** 10.1111/jce.16282

**Published:** 2024-04-23

**Authors:** Sahil Zaveri, Mohamed Chahine, Mohamed Boutjdir

**Affiliations:** 1 Cardiovascular Research Program, VA New York Harbor Healthcare System, New York, New York, USA; 2 Department of Medicine, SUNY Downstate Health Sciences University, New York, New York, USA; 3 CERVO Brain Research Center, Institut Universitaire en Santé Mentale de Québec, Québec City, Québec, Canada; 4 Department of Medicine, Faculté de Médecine, Université Laval, Quebec City, Québec, Canada; 5 Division of Cardiology, Department of Medicine, NYU Grossman School of Medicine, New York, New York, USA

**Keywords:** genetic variants, Hispanic, Indigenous, ion channelopathies, Latino, sudden cardiac death

## Abstract

The limited literature and increasing interest in studies on cardiac electrophysiology, explicitly focusing on cardiac ion channelopathies and sudden cardiac death in diverse populations, has prompted a comprehensive examination of existing research. Our review specifically targets Hispanic/Latino and Indigenous populations, which are often underrepresented in healthcare studies. This review encompasses investigations into genetic variants, epidemiology, etiologies, and clinical risk factors associated with arrhythmias in these demographic groups. The review explores the Hispanic paradox, a phenomenon linking healthcare outcomes to socioeconomic factors within Hispanic communities in the United States. Furthermore, it discusses studies exemplifying this observation in the context of arrhythmias and ion channelopathies in Hispanic populations. Current research also sheds light on disparities in overall healthcare quality in Indigenous populations. The available yet limited literature underscores the pressing need for more extensive and comprehensive research on cardiac ion channelopathies in Hispanic/Latino and Indigenous populations. Specifically, additional studies are essential to fully characterize pathogenic genetic variants, identify population-specific risk factors, and address health disparities to enhance the detection, prevention, and management of arrhythmias and sudden cardiac death in these demographic groups.

## 1 | INTRODUCTION

There is a growing interest in cardiac electrophysiology studies with a particular focus on cardiac ion channelopathies and sudden cardiac death (SCD) in diverse populations. Previous research studies have shown disparities in the management of arrhythmias and treatment effectiveness in various ethnic groups, including African American (AA)/Black and Asian populations, but only limited data exist for Hispanic/Latino and Indigenous groups.^1–3^ Moreover, health disparities that could impede the detection of these arrhythmias in these two populations have yet to be fully explored. The goal of this review is to advance our understanding of the fundamental aspects of cardiac pathophysiology and shed light on potential population-specific factors influencing susceptibility to SCD. This ultimately leads to targeted interventions and therapeutic strategies tailored to different populations at risk of cardiac arrhythmias and SCD.

## 2 | ADDRESSING DISPARITIES IN AFRICAN AMERICAN AND ASIAN POPULATIONS

We have recently examined racial disparities in the management of ion channelopathies and SCD in ethnically diverse populations.^2,3^ In one of these studies, Chahine et al. reported that AA/Black subjects were notably underrepresented in clinical trials on ion channelopathies and SCD.^3^ We highlighted limited genetic testing and the underuse of medical devices in managing these patients and proposed solutions to address these disparities, including early identification of genetic variants, diverse inclusion in trials, and tailored preventive management.^3^ A study by Zhao et al. found that socioeconomic and cardiovascular risk factors lead to approximately 65% excess SCD risk in AA/Black populations.^4^ The solutions proposed involve compliance with medical guidelines, facilitating referrals to cardiologists, and investing in the training and mentorship of underrepresented junior faculty in cardiovascular research to address the disparities in treating these patients.

A subsequent review by Zaveri et al. examined the racial and ethnic differences of Asian populations with ion channelopathies and SCD by compiling current research on cardiac ion channelopathies and genetic disorders.^2^ This study details the evolving understanding of SCD while examining differences in research, treatment, and outcomes between Asian and White populations, with an emphasis on Asian patients’ phenotypic abnormalities, device usage, and mortality risk. The review delves into the specific differences in SCD genetic risk factors in Asian populations compared to White populations.^2^ Similar to the study conducted by Chahine et al., Zaveri et al. proposed solutions such as early genetic screening, addressing barriers to medical care and device utilization, improving physician training, and enhancing patient education on risk factors.^2,3^

## 3 | GAP IN KNOWLEDGE: HISPANIC/ LATINO AND INDIGENOUS POPULATIONS

Herein, we aimed to conduct a review of ion channelopathies and SCD studies in two underrepresented demographics, namely Hispanics/Latinos and Indigenous populations. Surprisingly, we discovered that there is an immense deficit of studies available.^5^ This highlights a stark need for further studies on the genetic risk factors in these populations and the socioeconomic disparities that may increase the lifetime risk of SCD. Our aim with this review is to highlight the necessity of expanding the body of available medical research on adverse cardiovascular arrhythmic events and ion channelopathies in Hispanic/Latino and Indigenous demographics to eventually bridge disparities in the detection, prevention, and management of these conditions.

## 4 | DEFINING HISPANIC/LATINO AND INDIGENOUS POPULATIONS

It is crucial to differentiate between the terms Hispanic and Latino. Hispanic specifically pertains to individuals with ancestry from Spanish-speaking countries, whereas Latino broadly encompasses individuals with roots in Latin American regions, including Central America, South America, Mexico, and the Caribbean.^6^ Hispanics/Latinos are a unique population as, despite facing a growing number of healthcare disparities, they continue to be the fastest-growing ethnic/racial demographic in the United States.^6^ Similarly, it is crucial to distinguish among the terms Indigenous, Native American, First Nations, and Aboriginal. Indigenous is a more encompassing term, applicable globally, referring to the first peoples of various regions worldwide, emphasizing their shared cultural and ancestral connections.^7–9^ Native American, also known as American Indian, refers specifically to the Indigenous peoples of the United States, whereas First Nations often refers to Indigenous peoples of Canada. The term Aboriginal is commonly used to refer to the original inhabitants of Australia.^8,10–13^

## 5 | GENETIC STUDIES IN HISPANIC/LATINO POPULATIONS

There have been efforts to characterize specific ion channelopathy genetic variants in Hispanic and Latino populations. A genome-wide association study conducted by Mendez-Giraldez et al. examined QT interval prolongation in a Hispanic and Latino population, revealing six secondary signals at specific genes, including *NOS1AP*, *ATP1B1*, *SCN5A*, and *KCNQ1*.^14^ A comparison of linkage disequilibrium patterns suggested that lead single-nucleotide polymorphisms (SNPs) in *SCN5A* and *KCNE1* might be novel and specific to the Hispanic/ Latino populations.^14^ Another study conducted by Arking et al. on QT interval prolongation in the Hispanic/Latino demographic highlighted SNPs in *NOS1AP* that may be associated with an increase in QT interval.^15^ Nonetheless, it should be noted that this study did not reach statistical significance in the Hispanic and Latino groups.^15^ A study by Shah et al. further examined the link between *NOS1AP* and QT interval change in a multi-ethnic group and found potentially significant associations in the 5′ end of *NOS1AP* in the Hispanic cohort.^16^ In a fine-mapping study, Avery et al. discovered a significant association between QT interval prolongation and SNPs in *NOS1AP*, *SCN5A*, and *SCN10A* in Hispanic/Latino participants.^17^ Despite the association of *NOS1AP* with QT interval prolongation, it is important to note that *NOS1AP* does not encode an ion channel but rather a cytosolic protein that binds nitric oxide synthase. However, studies in a mouse model of dystrophic cardiomyopathy have demonstrated that *NOS1AP* shows a high degree of co-localization with L-type calcium channel and the inwardly rectifying potassium channel Kir3.1 suggesting functionally relevant interactions with the ion channels that regulate the action potential duration.^18^

In a recent cross-sectional observational study by Manini et al., the authors prospectively enrolled adult patients presenting to the emergency department with acute drug overdose secondary to prescription medications and illicit substances over a 2-year period.^19^ A noteworthy discovery from this analysis is the observation that individuals of Hispanic descent demonstrated a relative resistance to drug-induced QT prolongation in cases of overdose. This finding is of particular significance as it highlights a distinctive characteristic within the Hispanic population that renders them less susceptible to the prolongation of QT intervals caused by drug overdose. This resistance to drug-induced QT prolongation in Hispanic populations may have implications for both clinical practice as well as further research endeavors. Understanding this inherent resistance could potentially influence medical decision-making, treatment strategies, and dosage adjustments in the management of drug overdoses, particularly in Hispanic patients. Delving deeper into the molecular and genetic aspects of this resistance may unravel novel insights into the mechanisms underlying QT interval regulation, thus providing a foundation for future investigations and the development of targeted therapeutic interventions.^20^ These results suggest that race-specific factors may influence electrocardiogram (ECG) outcomes. Therefore, a detailed exploration of these factors is essential for a more accurate and inclusive interpretation of SCD and cardiac health assessments in general.

**FIGURE 1:**
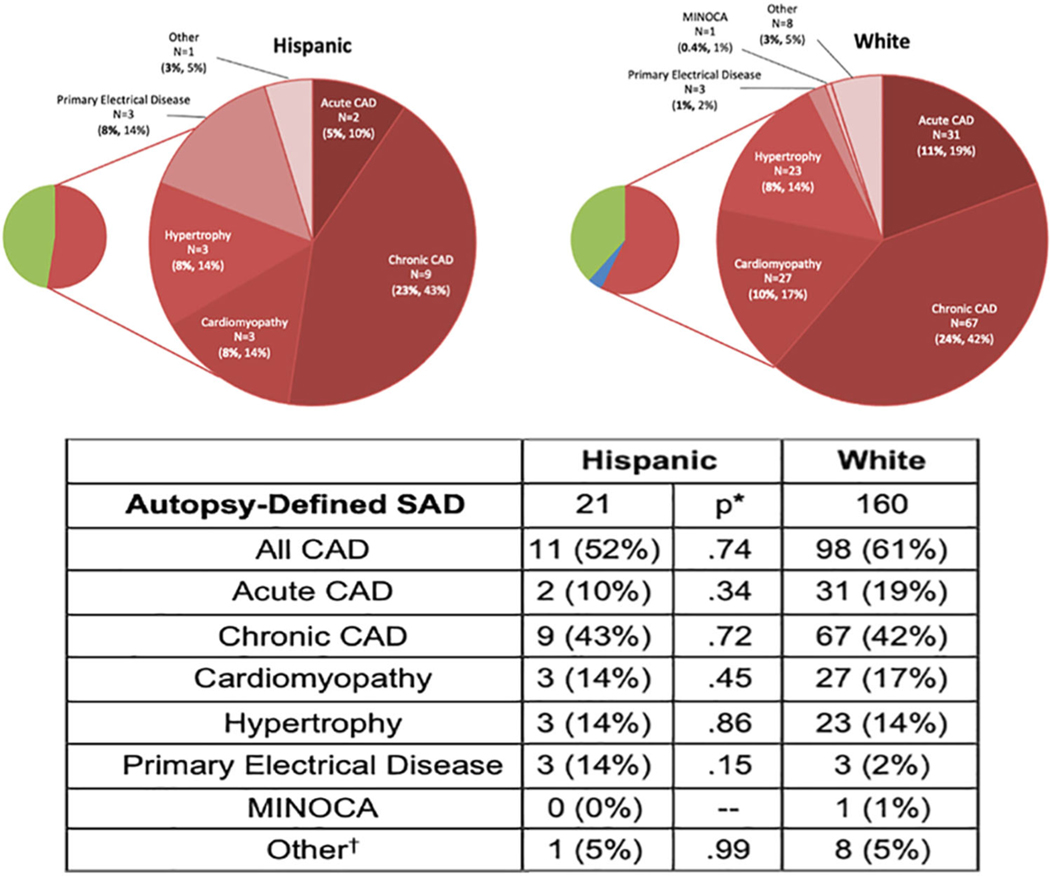
Ethnic disparities in autopsy-identified causes of sudden arrhythmic death. Illustration of autopsy-determined causes of sudden arrhythmic death (SAD) in Hispanic populations and reference White cohorts. Data reveal significant variations in SAD causes among Hispanic and White populations. For instance, Hispanics exhibit a higher prevalence of primary electrical disease as a cause of SAD compared to Whites (14% vs. 2%, *p* < .01). Reprinted from Tseng et al. with permission.^21^

As mentioned above, while genetic variants that contribute to arrhythmogenicity and racial disparities that increase the risk of SCD have been characterized in AA/Black and Asian demographics, albeit to a limited extent, there is a need to identify similar factors in Hispanic/Latino and Indigenous populations to fully characterize ion channelopathies and SCD risk profiles in these groups. In a study comparing autopsy-defined causes of sudden arrhythmic death (SAD) by Tseng et al., Hispanic subjects had a lower rate of developing SAD due to a fatal arrhythmia compared to White subjects (Figure 1).^21^ Of note, there was a higher incidence of Hispanic subjects who developed primary electrical disease compared to the reference White cohort.^21^

**FIGURE 2:**
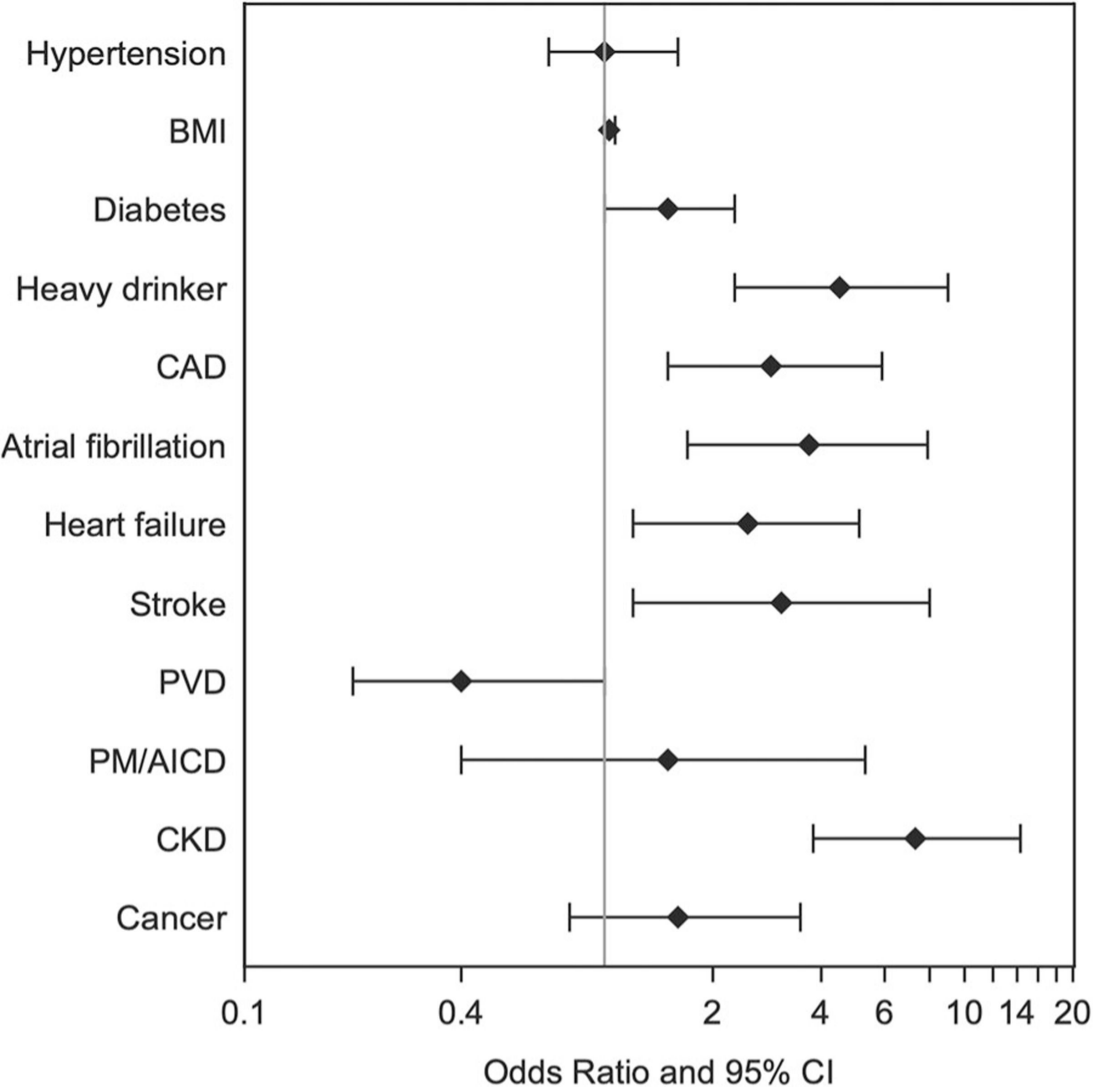
Major clinical determinants linked to sudden cardiac arrest in Hispanic/Latino populations. The odds ratios and 95% confidence intervals presented in this figure stem from a multivariable logistic regression model, which has been adjusted for age, sex, and all clinical variables depicted. Established cardiovascular diseases, including coronary artery disease, atrial fibrillation, heart failure, stroke, chronic kidney disease, and heavy alcohol consumption, are significantly associated with an increased risk of sudden cardiac arrest. Additionally, diabetes shows a marginal association with increased risk, whereas PVD exhibits a marginal association with decreased risk. BMI, body mass index; CAD, coronary artery disease; CKD, chronic kidney disease; PM/AICD, pacemaker/automated implanted cardioverter-defibrillator; PVD, peripheral vascular disease. Reprinted from Reinier et al. with permission.^22^

The available medical knowledge to date includes the PRESTO and HCHS/SOL study conducted by Reinier et al. to highlight risk factors for SCD in a Hispanic and Latino demographic.^22^ This study conducted in Ventura County, California, and the San Diego site of the Hispanic Community Health Survey/Study of Latinos is the first to assess predictors of SCD risk, specifically among Hispanic and Latino individuals in the United States. Analyzing data from 295 Hispanic and Latino SCD cases and 590 frequency-matched controls, the study identified several clinical variables associated with SCD (Figure 2).^22^ These associations held true even after adjusting for age, sex, and other clinical variables. A review by Kiernan et al. highlighted the disproportionate effect of SCD disease burden in non-Caucasian identifying populations and racial and ethnic differences in the efficacy of implantable cardioverter-defibrillators in these groups.^23^ In a retrospective postmortem study on sudden explained deaths, Lin et al. utilized high-resolution variant classification in cardiac arrhythmogenic gene testing within a diverse cohort.^24^ The study found that 3.1% of the Hispanic subjects tested positive for pathogenic or likely pathogenic arrhythmogenic genetic variants.^24^

In a 2015 study, Selga et al. characterized the genetic variation of Brugada syndrome (BrS) in a Spanish cohort, finding 19 variations in *SCN5A* that could potentially contribute to pathogenicity and also found that these variants could have a higher mean pathogenicity yield in a Spanish cohort compared to other European cohorts.^25^ Similarly, there are also select case studies highlighting ion channelopathies among Hispanic patients. One such case reported by Sharma et al. discussed BrS in a patient of Hispanic origin with ventricular fibrillation and an anomalous origin of the right coronary artery.^26^ The patient underwent successful intervention, and subsequent testing did not reproduce the Brugada pattern on an ECG. The case underscores the significance of thorough investigations and tailored management strategies in the context of such intricate cardiac presentations.^26^ Another case presented by Gautam et al. discussed the presentation of BrS masquerading as an acute coronary syndrome in a Hispanic patient, manifesting as an abnormal ST-segment elevation in the right precordial leads.^27^ This highlights the potential of BrS to cause SCD in patients with structurally normal hearts.

There have been efforts to characterize the global prevalence and clinical risk factors of cardiovascular disease (CVD) and arrhythmias in Hispanic/Latino populations. A key parameter has been improved management of atrial fibrillation (AF), the most prevalent arrhythmia globally affecting over 33 million individuals and projected to escalate by two to three times by 2050.^28^ The primary cause of death is ischemic heart disease, followed by cerebrovascular disease, collectively contributing to approximately half of all cardiovascular fatalities associated with AF.^28–30^ While AF is independently associated with an increased risk of SCD, it is imperative to differentiate between association and causation in this context. In individuals diagnosed with AF receiving anticoagulation therapy, SCD represents more than 20% of all deaths. Moreover, individuals with AF face a 2.5-fold elevated risk of SCD or ventricular fibrillation compared to those without AF.^28^

Both ion channel and non-ion channel variants have been found to be linked to AF.^31^ Among the ion channel variants associated with AF, Feghaly et al. have described several potassium channels, including *KCNQ1*, *KCNH2*, and *KCND3*, as well as sodium channels such as *SCN5A*, *SCN1B*, and *SCN2B*.^31^ Further supporting the role of ion channels in AF, Darbar et al. identified novel variants in the *SCN5A* gene in patients with AF, highlighting the potential contribution of these variants to arrhythmogenesis.^32^ The study underscores the importance of molecular phenotyping and targeted therapy in managing AF.^32^ Of note, while there have been reports of channelopathies in AF, most of the recent literature is focused on cardiomyopathic genetics.^33,34^ Therefore, while isolated ion channelopathies are recognized, they likely contribute to a lesser extent when compared with inherited cardiomyopathy syndromes.^33,34^ Recent studies have highlighted the significance of genetic testing in early-onset AF, demonstrating associations between rare variants in cardiomyopathy and arrhythmia genes with increased mortality risk and supporting the use of genetic testing in early-onset AF, particularly for prognostic purposes.^33,34^

A study by Linares et al. determined the weighted prevalence of AF in a representative Hispanic/Latino population (*n* = 16 415) to be 1%, with the highest prevalence in Dominicans (1.9%) and Puerto Ricans (2.5%) and the lowest prevalence in Mexicans (0.3%).^35^ Factors associated with higher AF prevalence included diabetes, hypertension, renal disease, left ventricular hypertrophy, and alcohol use.^36^ Interestingly, the association with renal disease is similar to the finding from the PRESTO, HCHS/SOL study conducted by Reinier et al. in Ventura Country, California.^22,37^ A retrospective study by Shulman et al. compared the predictive power of PR interval in the development of AF in non-Hispanic White, Hispanic, and AA/Black populations.^38^ The group discovered that at a PR interval of 196–201 ms, there was a significant association among all ethnic groups in the study. However, there was no significant association at PR intervals far above 200 ms.^38^

## 6 | THE HISPANIC PARADOX AND LIFESTYLE FACTORS

There is a unique epidemiological phenomenon called the Hispanic paradox, which states that Hispanics in the United States have a lower rate of CVD mortality and a longer life expectancy relative to non-Hispanic White counterparts despite a higher prevalence of CVD risk factors and disadvantageous socioeconomic conditions.^39^ This observation could guide further research on potential cardioprotective factors in Hispanic populations that could be extrapolated to other ethnicities. A systematic review and meta-analysis conducted by Ruiz et al. illustrated this paradox by examining 58 studies that reported on Hispanic all-cause mortality compared to those from other ethnicities.^6^ Overall, Hispanic populations had an overall lower risk of mortality compared to non-Hispanic White demographics and non-Hispanic Black demographics, and this variation was especially pronounced in the context of CVD. Another study by Mossavar-Rahmani et al. examined the prospective link between physical activity, sedentary behavior, and cardiometabolic biomarkers in a cohort of 8049 US Hispanics/Latinos.^40^ They revealed that low sedentary behavior and adherence to physical activity guidelines are linked to health benefits, especially in normoglycemic adults without CVD. This could further provide evidence for this paradoxical observation.^40^

While the link between channelopathies and arrhythmias is well-established, a growing body of research suggests that lifestyle modifications could have a more cardioprotective effect on channelopathies than previously hypothesized.^41^ For instance, studies have shown that regular aerobic exercise training can improve left ventricular function and reduce arrhythmic burden in patients with AF.^42^ Additionally, maintaining a healthy diet low in salt and rich in fruits and vegetables can help regulate electrolytes, particularly potassium and magnesium, which play a crucial role in electrical cardiac function and can be disrupted in some channelopathies.^43^ Furthermore, adequate sleep and stress management techniques have also been linked to a decreased risk of arrhythmic events, potentially by modulating autonomic nervous system activity, which in turn regulates ion channel functions.^44^ These findings highlight the potential for a more comprehensive approach to managing channelopathies, one that integrates traditional pharmacological interventions with targeted lifestyle modifications. This is particularly relevant for underrepresented groups such as Hispanic/Latino populations, where the prevalence of channelopathies may be underrecognized. Although these studies encompass various populations, including Hispanic/Latino communities, the collective body of evidence has substantially enriched our understanding of ion channelopathies and their prevalence of arrhythmias. Nonetheless, further studies should be conducted within families and various ethnic subgroups to determine significant genetic and environmental factors affecting the development of cardiac ion channelopathies in these populations.

## 7 | GENETIC STUDIES IN INDIGENOUS POPULATIONS

**FIGURE 3:**
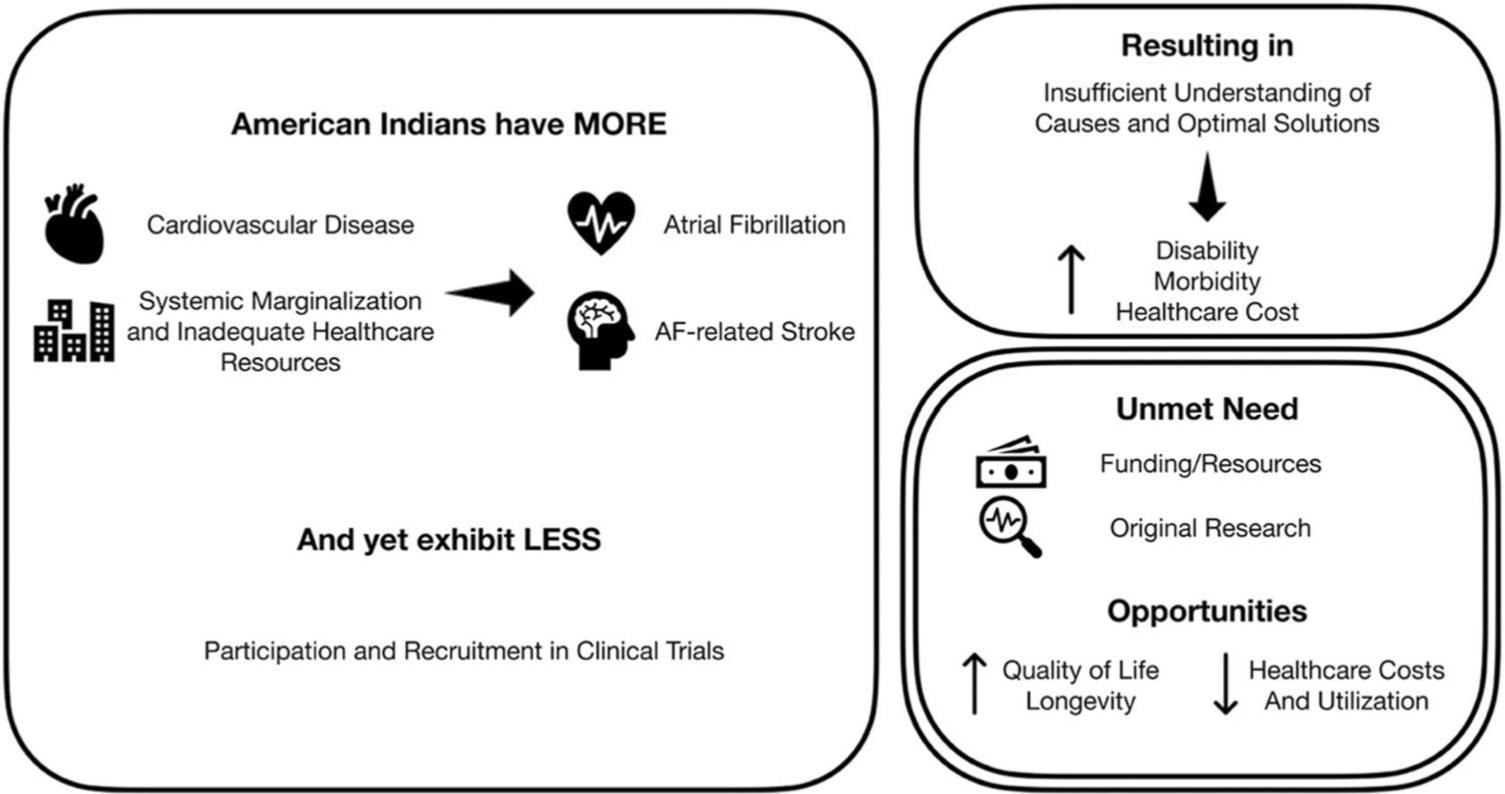
Cardiovascular disease disparities in American Indian populations. This figure highlights the complex interplay of factors contributing to increased cardiovascular disease incidence among American Indians. Factors such as systemic marginalization, inadequate healthcare resources, and heightened susceptibility to atrial fibrillation (AF) and AF-related strokes are depicted. Despite these challenges, American Indians exhibit lower rates of participation and recruitment in clinical trials, emphasizing the need for targeted interventions and improved access to healthcare within this demographic. AF, atrial fibrillation. Reprinted from Sanchez et al. with permission.^45^

Relative to the Hispanic and Latino demographics, there is an even greater need for further research into the effects of cardiac ion channelopathies in Native American and Aboriginal populations. In a 2015 review, Arbour et al. noted that some Inuit and First Nations communities in Canada exhibit higher rates of congenital heart malformations and long QT syndrome (LQTS), highlighting the relevance of genetic predispositions in specific groups of Aboriginals.^12^ A more recent 2023 population-based cohort study by Eberly et al. involved 220 598 American Indian and Alaska Native Medicare beneficiaries with a median age of 72.5 years and found that the prevalence of AF in this population was 9%.^11^ A systematic scoping review published in 2015 by Katzenellenbogen et al. examined the contemporary studies on AF epidemiology in the Indigenous populations of Australia, the United States, and New Zealand but found no clear patterns in the prevalence and incidence of AF among these populations.^9^ In a 2022 study, Sanchez et al. discussed that American Indians develop AF at a higher rate relative to other racial and ethnic groups, and there are also disparities in administering anticoagulation therapy, rhythm control strategies, and overall quality of healthcare in this population (Figure 3).^45^ Similar findings regarding the prevalence of AF were also demonstrated in a cross-sectional study of Indigenous Australians compared to non-Indigenous Australians.^46^ A 2021 study on implementing AF screening in a tribal primary care clinic using a mobile single-lead ECG device showed that among 1019 screened patients, new AF was diagnosed in 1.5% compared to 0.3% in the control group that did not undergo screening. The mean difference was 1.2%, indicating that mobile ECG screening detected a significantly higher incidence of AF than usual care, displaying the efficacy of implementing a deliberate AF screening protocol in Native American healthcare.^47^

There have also been a few research efforts to elucidate the genetic variants predisposing Native Americans and Aboriginal communities to ion channelopathies. Historically, there has been a paucity of data on Indigenous populations in large genomic datasets such as the Genome Aggregation Database.^8^ Furthermore, it is apparent that Indigenous populations have not yet experienced the benefits of technological advancements that aid genomic research and the diagnosis of rare diseases, including CVD.^7^ These disparities are even more pronounced in the research of ion channelopathies in these populations despite ongoing efforts to address these gaps. Swayne et al. identified a novel *ANK2* variant in multigenerational families, the carriers of which showed LQTS and exhibited signs of structural heart disease, including one with cardiomyopathy resulting in SCD.^48^ Two other studies highlighted the same missense mutation in the *KCNQ1* (V205M) associated with hereditary LQTS in First Nations subgroups in Northern British Columbia.^49,50^ The V205M variant showed variable effect in clinical expression, as 30% of the mutation carriers still had a corrected QT interval under 440 ms, showing the heterogeneous nature of hereditary LQTS, especially in this specific demographic. While these studies add significantly to the growing knowledge of ion channelopathies in Native American and Indigenous populations, further research is needed to adequately characterize these arrhythmogenic conditions from genetic factors to the overall clinical management in these underserved populations.

## 8 | FUTURE DIRECTIONS

Our examination of existing medical literature on ion channelopathies and their impact on Hispanic/Latino and Indigenous populations indicates that international and interinstitutional studies have identified genetic and environmental factors in these groups (Table 1). However, there is a need for additional research to establish a comprehensive consensus on the effects of genetic variants across various ethnic subgroups. This understanding can contribute to more targeted clinical treatments. For example, genome-wide association studies in Native American and Aboriginal communities could highlight SNPs in LQTS or other ion channelopathies, as was illustrated in the Hispanic community. Conversely, multigenerational studies conducted in Hispanic/ Latino communities could provide a more complete picture of hereditary LQTS. The Hispanic/Latino and Indigenous demographics comprise a significant portion of the general population, and as such, more extensive research studies have to be conducted to bridge healthcare disparities surrounding cardiac ion channelopathies in these underserved populations.

## 9 | CONCLUSION

The exploration of cardiac ion channelopathies and SCD across diverse populations has illuminated significant disparities in health-care access, treatment effectiveness, and genetic understanding. Studies focusing on AA and Asian populations have revealed underrepresentation in clinical trials and socioeconomic factors contributing to increased SCD risk. Similarly, the dearth of research in Hispanic/Latino and Indigenous populations underscores the urgent need for comprehensive studies to address genetic risk factors and socioeconomic disparities affecting SCD susceptibility. Despite the challenges, recent investigations have shed light on the prevalence and risk factors of arrhythmias in Hispanic/Latino and Indigenous communities, offering valuable insights into potential cardioprotective factors and genetic variants associated with these conditions. Moving forward, collaborative efforts involving international and interinstitutional studies are essential to establish consensus on genetic influences and develop targeted interventions tailored to various ethnic subgroups. By prioritizing research in these underserved populations, significant progress towards bridging healthcare disparities and improving outcomes for individuals affected by cardiac ion channelopathies across diverse ethnicities can be achieved.

## Figures and Tables

**TABLE 1:** Summary of published literature on Hispanic/Latino and Indigenous populations with cardiac ion channelopathies and sudden cardiac death.

Study name	Study design	Study outcome
Risk factors for sudden cardiac arrest among Hispanic or Latino adults in Southern California: Ventura PRESTO and HCHS/SOL^22^	Case–control study	Chronic kidney disease was the top predictor of sudden cardiac arrest risk in Hispanic adult subjects in this study
Comprehensive genetic characterization of a Spanish Brugada syndrome cohort^25^	Retrospective cohort study	This study identified 14 Brugada syndrome-susceptibility genes in a Spanish cohort, revealing a higher pathogenic variation discovery yield (32.7%) compared to other European BrS cohorts
Ethnic differences in genetic ion channelopathies associated with sudden cardiac death: a systematic review and meta-analysis^14^	Systematic review and meta-analysis	There are significant ethnic variations in alleles associated with SCD, with Asians having the highest mean allele frequencies for *NOS1AP* and *SCN5A*, Caucasians for *KCNH2*, and Hispanics for *KCNQ1*
GWAS of the electrocardiographic QT interval in Hispanics/Latinos generalizes previously identified loci and identifies population-specific signals^15^	Meta-analysis	This study identified 41 genome-wide significant SNPs across 13 known QT loci and revealed potential novel, population-specific variants at *SCN5A* and *KCNE1*
Multiple independent genetic factors at NOS1AP modulate the QT interval in a multi-ethnic population^16^	Retrospective cohort study	This study on genetic variants in *NOS1AP* with QT interval to a multi-ethnic population reveals a significant association in Whites and Blacks and a potential association in Hispanics
Associations between NOS1AP single nucleotide polymorphisms (SNPs) and QT interval duration in four racial/ethnic groups in the Multi-Ethnic Study of Atherosclerosis (MESA)^17^	Retrospective cohort study	The study explored the effects of *NOS1AP* variants on QT interval duration in a diverse cohort, finding stronger associations in Caucasians, weaker evidence in Hispanics and Chinese, and potential novel associations at the 3′ end of *NOS1AP* in Chinese participants
Cardiac arrest with “pseudo-Brugada” ECG pattern in the setting of a coronary artery anomaly^26^	Case report and literature review	The study describes a case of a Hispanic man with ventricular fibrillation and BrS, whose anomalous origin of the right coronary artery was surgically corrected, leading to the normalization of the Brugada ECG pattern
A case of Brugada syndrome masquerading as acute coronary syndrome in a Hispanic male^27^	Case report	This case report described the management of a 58-year-old male with BrS manifesting similarly to acute coronary syndrome after ruling out structural heart diseases
Prevalence of atrial fibrillation and association with clinical, sociocultural, and ancestral correlates among Hispanic/Latinos: The Hispanic Community Health Study/Study of Latinos^35^	Population-based prospective cohort study	The study found a low overall AF prevalence (1.0%) in a diverse Hispanic/Latino population, indicating varying risks across ethnic backgrounds, with individuals of Dominican and Puerto Rican heritage having higher AF risks than those of Mexican background
Evaluation of sudden cardiac arrest by race/ethnicity among residents of Ventura County, California, 2015–2020^37^	Prospective cohort study	The study found that the burden of sudden cardiac arrest was similar in Hispanic and White cohorts and lower in Asian cohort and identified shared SCA risk factors among Asian and Hispanic populations
Validation of PR interval length as a criterion for development of atrial fibrillation in non-Hispanic whites, African Americans and Hispanics^38^	Retrospective epidemiological study	The study validates a PR interval of 200ms as a criterion for predicting AF in AA and Hispanics, but suggests that this value may be less sensitive in predicting AF in AA compared to non-Hispanic Whites
Are sedentary behavior and physical activity independently associated with cardiometabolic benefits? The Hispanic Community Health Study/Study of Latinos^40^	Prospective cohort study	Hispanics/Latinos without cardiometabolic disease and those who met guidelines for low-to-moderate physical activity experienced a significant decline in LDL-cholesterol and lower fasting glucose levels indicating a population-specific benefit of lifestyle determinants of health
Confronting the growing crisis of cardiovascular disease and heart health among Aboriginal peoples in Canada^13^	Literature review	This literature review article discusses the increasing burden of CVD among Canadian Aboriginal peoples, attributed to dietary changes and reduced physical activity
The genetics of cardiovascular disease in Canadian and International Aboriginal Populations^12^	Literature review	This study emphasizes the genetic factors influencing CVD in Aboriginal populations, noting distinctions in disease rates, including higher incidences of congenital heart malformations and LQTS in specific communities
Cardiovascular disease burden and outcomes among American Indian and Alaska native Medicare beneficiaries^11^	Population-based cohort study	This study discusses the significant burden of CVD and cardiometabolic risk factors among American Indian and Alaska Native Medicare beneficiaries
American Indians and atrial fibrillation^45^	Literature review	This study discusses the heightened risk of AF and AF-related stroke in the American Indian population
Atrial fibrillation in Indigenous and non-Indigenous Australians: a cross-sectional study^46^	Retrospective cross-sectional study	This study discusses the age-dependent prevalence of AF in Indigenous and non-Indigenous Australians, highlighting significantly higher rates among young Indigenous Australians
Screening for atrial fibrillation in American Indian adults in a tribal primary care clinic^47^	Prospective cohort study	This study demonstrates the feasibility and efficacy of opportunistic, mobile single-lead ECG screening for AF in American Indian adults, revealing a higher prevalence of AF than in the usual care group, especially among those under 65, emphasizing the potential benefits of earlier AF screening in this population
Comparison of QRS duration and associated cardiovascular events in American Indian men versus Women (The Strong Heart Study)^48^	Prospective cohort study	This cohort study compares American Indian men and women, showing that elevated QRS duration is an independent predictor of CVD in women, particularly associated with higher risks of coronary heart disease and myocardial infarction
A KCNQ1 V205M missense mutation causes a high rate of long QT syndrome in a First Nations community of northern British Columbia: a community-based approach to understanding the impact^49^	Comparative study	This study, conducted in a remote Canadian First Nations community, identifies a novel *KCNQ1* mutation (V205M) associated with hereditary LQTS, leading to variable clinical expression

Abbreviations: AA, African American; AF, atrial fibrillation; BrS, Brugada syndrome; CVD, cardiovascular disease; ECG, electrocardiogram; LQTS, long QT syndrome; SCD, sudden cardiac death; SNPs, single-nucleotide polymorphisms.

## Data Availability

The data that support the findings of this study are available from the corresponding author upon reasonable request.

## Abbreviations:

AA: African American
AF: atrial fibrillation
BrS: Brugada syndrome
CVD: cardiovascular disease
ECG: electrocardiogram
LQTS: long QT syndrome
SAD: sudden arrhythmic death
SCD: sudden cardiac death
SNPs: single-nucleotide polymorphisms
